# A Community-Wide Collaboration to Reduce Cardiovascular Disease Risk: The Hearts of Sonoma County Initiative

**DOI:** 10.5888/pcd16.180596

**Published:** 2019-07-11

**Authors:** Allen Cheadle, Michelle Rosaschi, Dolores Burden, Monica Ferguson, Bo Greaves, Lori Houston, Jennifer McClendon, Jerome Minkoff, Maggie Jones, Pam Schwartz, Jean Nudelman, Mary Maddux-Gonzalez

**Affiliations:** 1 Center for Community Health and Evaluation, Kaiser Permanente Washington Health Research Institute, Seattle, Washington; 2 Redwood Community Health Coalition, Petaluma, California; 3 Kaiser Permanente Medical Group, Sonoma County, California; 4 St. Joseph Health Medical Group, Irvine, California; 5 Vista Family Health Center, Vista, California; 6 Sonoma County Department of Health Services, Santa Rosa, California; 7 Northern California Center for Well-Being, Santa Rosa, California; 8 Kaiser Permanente, Oakland, California

## Abstract

**Purpose and Objectives:**

Collaboration across multiple sectors is needed to bring about health system transformation, but creating effective and sustainable collaboratives is challenging. We describe outcomes and lessons learned from the Hearts of Sonoma County (HSC) initiative, a successful multi-sector collaborative effort to reduce cardiovascular disease (CVD) risk in Sonoma County, California.

**Intervention Approach:**

HSC works in both clinical systems and communities to reduce CVD risk. The initiative grew out of a longer-term county-wide collaborative effort known as Health Action. The clinical component involves activating primary care providers around management of CVD risk factors; community activities include community health workers conducting blood pressure screenings and a local heart disease prevention campaign.

**Evaluation Methods:**

The impact of the clinical improvement efforts was tracked using blood pressure data from the 4 health systems participating in HSC. Descriptive information on the community-engagement efforts was obtained from program records. Lessons learned in developing and maintaining the collaborative were gathered through document review and interviews with key informants.

**Results:**

Favorable trends were seen in blood pressure control among patients with hypertension in the participating health systems: patients with controlled blood pressure increased from 58% in 2014 to 67% in 2016 (*P* < .001). Between 2017 and 2019, the community engagement effort conducted 99 outreach events, reaching 1,751 individuals, and conducted 1,729 blood pressure screenings, with 441 individuals referred to clinical providers for follow-up care. HSC scored highly on 6 essential elements of an effective coalition and achieved a degree of sustainability that has eluded many other collaboratives.

**Implications for Public Health:**

Factors contributing to the success of HSC include 1) starting small and focused to build trust among participants and demonstrate value, 2) working within the framework of a larger effort, and 3) providing long-term, open-ended backbone support.

**Summary**

**What is already known on this topic?**

Clinical and community collaborations are foundational to primary care transformation efforts, but it has proved challenging to build sustainable, effective collaborations.

**What is added by this report?**

Several lessons from the experience of the successful Hearts of Sonoma County (HSC) collaborative, including 1) start small and focused to build trust among participants and demonstrate value, 2) work within the framework of a larger effort, and 3) providing long-term, open-ended backbone support.

**What are the implications for public health practice?**

The HSC experience may provide a roadmap for other, similar efforts.

## Introduction

Improving the health of a population requires a multi-faceted approach that includes both community and clinical strategies (1). Implementing these clinic/community strategies successfully often requires multi-sectoral collaborations that bring together a broader range of organizations and institutions than are part of typical public health coalitions (2). For example, multi-sector Accountable Communities of Health have been part of many State Innovation Model (3) health improvement projects that are attempting to bring together a range of partners to work on health system transformation (4).

Although effective collaboration is needed to bring about health system transformation, doing it well has proved challenging. In a recent study by Siegel et al (5) of 145 health system improvement collaboratives that had a reputation for being mature and effective, as few as 10 were judged to be mature enough to make true progress toward supporting a transformed health system. Some of the challenges that have limited the effectiveness of previous public health–oriented coalitions (6) are accentuated in these newer, larger collaboratives encompassing more sectors (ie, reaching agreement on goals, approaches, and steps to action among varied organizations with competing organizational objectives).

One way of overcoming these challenges is to learn from successful collaborative efforts. Substantial literature on what makes a successful coalition exists (2,7,8), but we are aware of few published examples in which multi-sector collaborative efforts have been sustained over an extended period, and long-term sustainability is critical for creating a transformed, integrated health care system.

In this article, we describe the Hearts of Sonoma County (HSC) initiative, a county-wide, multi-sector collaborative effort to reduce cardiovascular disease (CVD) risk in Sonoma County, a medium-sized county in northern California. HSC grew out of Health Action, a larger multi-sector effort that has existed for more than 10 years. HSC is being evaluated using 1) a process evaluation to capture milestones in initiative development and factors associated with success, and 2) an outcome evaluation documenting changes in CVD outcomes (eg, blood pressure control) using pooled county-level provider data. This article describes the initiative and outcomes to date and identifies lessons learned and recommendations that may be useful for other, similar initiatives.

## Purpose and Objectives

Sonoma County is the northwestern-most county in the 9-county San Francisco Bay Area region, with a population of 502,000 in in 2016 (9). Its county seat and largest city is Santa Rosa. The county is near the average for California in terms of income/poverty: the median household income of $61,000 is below the $67,700 statewide median, but the federal poverty rate is lower than the state as a whole — 11.2% versus 14.3% (9). The largest racial/ethnic groups are white (66%) and Hispanic (25%) (9). From 2015 through 2017, 31% of adults in Sonoma County had ever been diagnosed with high blood pressure, and 7% had ever been diagnosed with heart disease (10). Health care providers include Kaiser Permanente, St. Joseph Health, Sutter Health, and several federally qualified community health centers.

In 2007 the Sonoma County Department of Health Services, which includes the public health department, approached the county board of supervisors with a proposal to form a collaborative to address social determinants of health and health equity. Health care was at the top of the county agenda because of a public hospital closing, and there was a growing recognition that health involves more than just health care. Therefore, the board of supervisors adopted the proposal and formed the Health Action collaborative.

Health Action brought together organizations in education, business, health care, labor, and public health to focus on social determinants of health and health equity and justice. Three focus areas were chosen: health care, education, and economic wellness, and standing committees created in each area. Education, known as Cradle To Career, focused on educational and social strategies to support children and youth reaching their fullest potential at every stage of life, such as coordinating a campaign focused on school attendance to address the effect of student absenteeism and working to develop agreed-upon local standards for college and career readiness in Sonoma County. Economic wellness focused on addressing local economic conditions and issues to support families becoming better able to make ends meet, such as affordable housing and helping low-income families take advantage of the earned income tax credit. The health care committee (the Committee for Healthcare Improvement) focused initially on primary care, addressing a shortage of primary care physicians and working to increase Patient-Centered Medical Home (PCMH) capacity. Over time, the committee recognized that a broadened focus was needed and shifted their attention first to end-of-life care and then, after a community health needs assessment, to reduce CVD risk. The initial collaborations around assessing local primary care capacity and PCMH were critical in establishing trust across health care entities in a competitive market. This trust extended to sharing workforce data.

HSC was formed by the committee in 2014 as a result of the new focus on CVD risk reduction. Drawing from the Centers for Disease Control and Prevention’s Million Hearts campaign (11) and work being done by Kaiser Permanente to implement an effective algorithm for reducing CVD risk (12), individual provider organizations participating in the committee began implementing improved practices in their clinics in 2014. The fact that the community health centers and Kaiser Permanente groups served more than half of the population and were both engaged in cardiovascular health initiatives was a major factor in deciding to focus on CVD across health systems.

In 2016 the county applied for and received a California Accountable Communities of Health Initiative (CACHI) grant to transform and operationalize the work of Health Action by piloting accountable communities of health principles to address CVD with HSC through health care system and community-based interventions. Community outreach, education, and engagement efforts coalesced in the It’s Up to Us campaign, a partnership between the United Way of the Wine Country and the Northern California Center for Well-Being (Center for Well-Being), which was launched in 2017. It’s Up to Us has 3 primary goals: 1) educate the community about CVD risk factors, 2) conduct community-based blood pressure screenings, and 3) link high-risk individuals to primary care to reduce risk of heart attacks and strokes.

## Intervention Approach

**Figure 1:**
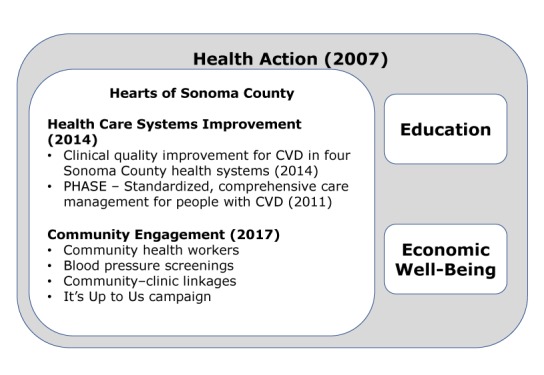
Sonoma County Health Action Collaborative, overall structure and health care activities, the Hearts of Sonoma County Initiative, Sonoma County, California. Abbreviations: CVD, cardiovascular disease; PHASE, Preventing Heart Attacks and Strokes Everyday.

This section describes the clinical care and community engagement components of HSC, as well as the structure, and operations of the HSC collaborative. Figure 1 shows the structure of HSC and its position within Health Action. 

### Improving clinical care

The goal of the clinical care effort is to activate primary care providers around evidence-based interventions, including improved identification and management of hypertension and more consistent screening for other CVD risk factors, coupled with more robust smoking cessation support. With the funding from CACHI, the scope of the clinical effort was expanded to include secondary prevention modeled on the Kaiser Permanente Preventing Heart Attacks and Strokes Everyday (PHASE) initiative, which encompasses standardized, comprehensive care management and cardio-protective medications for people with CVD and those who have had a heart attack or stroke. The PHASE strategies being implemented include adoption of evidence-based clinical guidelines and standardized procedures for registered nurses; capacity building for population health management; provider/clinician/medical staff education and training; primary care workflow improvements; and extended team-based care. The population health framework introduced through PHASE is used by each entity, wherein the population at risk is identified and stratified and interventions and results are tracked on the population as a whole and by individual providers and, in some cases, by care team staff. This approach is effective in influencing clinical practice and improving outcomes.

### Community engagement

The second part of the HSC strategy was to engage the community around CVD risk reduction and help link efforts in the clinical domain with interventions across the community, policy, systems, and environmental domains. Activities have included convening a new Community Engagement workgroup, training community health workers (CHWs) to conduct community-based education and blood pressure screenings, and convening a media workgroup to partner in a localized heart disease prevention media campaign. The following provide a brief summary of those activities.

**Community Engagement workgroup**. A Community Engagement workgroup facilitated by Center for Well-Being staff planned and implemented the campaign, including listening sessions (15 listening sessions, engaging 170 participants) to ensure the subsequent campaign spoke to populations at greatest risk for heart disease. Community was integral in shaping campaign messaging to shift their perception of risk and motivate them to take action.

**Community-based education and blood pressure screenings.** The Center for Well-Being developed a training module for Promotores de Salud/CHWs to be trained in blood pressure screening, identifying risk factors and warning signs, and learning what to do when they encounter residents with blood pressure outside the normal range, including how to link residents to care. Once trained, the Center for Well-Being leveraged existing partnerships to begin outreach in nontraditional settings. The Center for Well-Being developed a protocol to contact community members found to have high (140–169 mm Hg systolic or 90–99 mm Hg diastolic) or very high (≥170 mm Hg systolic or ≥100 mm Hg diastolic) blood pressure readings a few days after the screening to learn if they followed through with scheduling an appointment with their medical provider or contacting a clinic if they were out of care. The Center for Well-Being made arrangements with one community health center site in Santa Rosa, enabling CHWs to use a direct phone line to schedule medical appointments for people as soon as possible. Center for Well-Being staff links residents to additional support services, including health insurance assistance and behavioral change classes to prevent heart disease.

**Localized heart disease prevention campaign**. Listening session results were developed into 3 campaign concepts, further tested with residents from our target populations and revised based on their feedback. The goal of the It’s Up to Us campaign, launched in August 2017, is community empowerment, encouraging people to take ownership of their health, with a first action of checking their blood pressure. Images, taglines, and the corresponding website (CheckYourBP.org) provide a cohesive media and messaging campaign. Collateral material such as the blood pressure cards and posters were designed and distributed to health care partners.

### HSC collaborative identity and functioning

The HSC collaborative has evolved over time, from starting as an initiative of the Committee for Healthcare Improvement (a committee of the larger Health Action collaborative) to piloting how Health Action will function as an Accountable Community for Health. Table 1 lists the HSC partner organizations and their role on the project, as defined by their membership in workgroups and committees. Table 2 lists these same organizations and shows which parts of the organization are represented regularly at meetings (eg, clinical representatives, organizational leadership, administration/program managers).

**Table 1:** Partner Organization Participation in Key Initiative Components, Hearts of Sonoma County Initiative, Sonoma County, California

Organization	Type of Organization	Health Action Council	Committee for Healthcare Improvement	HSC Leadership Team	HSC Membership	HSC Clinical WG	HSC Community-Clinical Linkages WG	HSC Community Engagement WG	HSC Data WG and Participation in Data Sharing	Accountable Communities of Health Oversight Committee
Center for Well-Being	Community-based organization: wellness programs	X		X		X	X	X		X
Ceres Community Project	Community-based organization: nutrition/meal assistance			X		X	X	X		
Farm To Pantry	Community-based organization: nutrition/meal assistance				X		X			
Health Action	Multi-sector collaborative	X	X	X	X	X	X	X	X	X
Integrative Medical Clinic Foundation	Health care provider				X	X	X	X		
Kaiser Permanente Santa Rosa	Health care provider				X	X	X		X	
Northern California Medical Associates	Local medical association									
Partnership HealthPlan of California	Managed Medicaid health plan		X		X	X	X			X
Petaluma Health Care District	Public health district	X			X	X	X	X		
Redwood Community Health Coalition	Consortium of community health centers	X	X		X	X	X	X	X	X
Santa Rosa Family Medicine Residency	Health care provider				X	X	X			
Santa Rosa Community Health	Health care provider	X	X		X	X	X	X	X	X
Sonoma County Department of Health Services	County public health department	X	X	X	X	X	X	X	X	X
Sonoma County Department of Health Services Tobacco Team/Smoke Free Tobacco Coalition	County public health department				X	X	X	X		
Sonoma County Family YMCA	Community-based organization: wellness programs				X	X	X	X		
St Joseph Health Medical Group	Health care provider	X	X		X	X	X		X	
St. Joseph Health Sonoma County, Health Promotion Programs	Health care provider				X	X	X	X		
Sutter Medical Group of the Redwoods	Health care provider	X	X		X	X	X		X	
The Permanente Medical Group/Kaiser Permanente Santa Rosa	Health care provider	X	X		X	X	X		X	
United Way of the Wine Country	Non-profit organization/fundraising coalition				X			X		
West County Health Centers	Health care provider			X	X	X	X		X	

Abbreviations: HSC, Hearts of Sonoma County; WG, workgroup.

**Table 2:** Organizational Staff, by Partner Organization, Hearts of Sonoma County Initiative, Sonoma County, California

Organization	Clinical Representation^a^	Quality/Data Representation	Leadership Representation	CHW Participation	Administration/Program Managers
Center for Well-Being			X	X	X
Ceres Community Project			X		X
Farm To Pantry			X		
Health Action	X		X		
Integrative Medical Clinic Foundation	X		X		X
Kaiser Permanente Santa Rosa	X	X	X		
Northern California Medical Associates	X				
Partnership HealthPlan of California	X		X		
Petaluma Health Care District			X		
Redwood Community Health Coalition	X	X	X		X
Santa Rosa Family Medicine Residency	X		X		X
Santa Rosa Community Health	X	X	X		X
Sonoma County Department of Health Services		X	X		
Sonoma County Department of Health Services Tobacco Team/Smoke Free Tobacco Coalition			X		X
Sonoma County Family YMCA			X		X
St Joseph Health Medical Group	X	X	X		X
St Joseph Health Sonoma County, Health Promotion Programs		X	X	X	
Sutter Medical Group of the Redwoods	X	X	X		X
The Permanente Medical Group/Kaiser Permanente Santa Rosa	X	X	X		
United Way of the Wine Country			X		X
West County Health Centers	X	X	X		X

Abbreviation: CHW, community health worker.

a Includes providers, pharmacists, and nurses.

The Sonoma County Department of Health Services provides backbone support for HSC, and the It’s Up to Us community engagement work is backboned by the Center for Well-Being with funding from United Way. The Department of Health Services provides approximately 20 to 30 hours per week or 75% of a full-time position to coordinate HSC associated meetings. On average, the Center for Well-Being estimates 16 hours per week on work associated with HSC. However during the height of the campaign initiation (2017–2018), it was closer to 25 hours per week on campaign planning, coordination, and evaluation.

In 2018, HSC members assumed oversight of the CACHI Portfolio of Interventions, which includes management of mutually reinforcing clinical and community-based strategies that support the overall goal of improving cardiovascular health throughout Sonoma County. The clinical improvement and community engagement tracks operate independently but inform each other’s activities, with several operational connections now, including a Clinical–Community Linkages workgroup. For example, patients identified with high blood pressure at the community screenings are linked to health care providers who are represented on the Committee for Healthcare Improvement.

## Evaluation Methods

The evaluation design was largely retrospective and descriptive, documenting the development of the HSC initiative and its impact to the extent possible, given that this was not designed as a prospective research/evaluation study. The evaluation of HSC includes 1) documenting clinical care improvement efforts around CVD and the impact those changes have had on CVD outcomes; 2) capturing diverse community engagement efforts and their impact; and 3) working to understand the factors associated with the success of the collaborative, including challenges and lessons learned. The following is a brief description of the methods used in each of these three areas.

The long-term evaluation of the HSC clinical work is focused on tracking county-level CVD outcomes. HSC representatives recognized early the importance of sharing data, both for the continuous improvement and to document county-level outcomes. HSC clinical partner organizations signed a multi-party data sharing/nondisclosure agreement that enables them to report and aggregate data related to CVD risk factor interventions. To date, reporting partners have shared their Healthcare Effectiveness Data and Information Set (HEDIS) blood pressure control data annually to create a countywide report card that benchmarks and tracks how the local health system is doing overall with screening, diagnosing, and managing hypertension, and to track collective improvement. As of September 2017, 4 major primary care provider organizations in Sonoma County contributed 2014, 2015, and 2016 numerator and denominator totals for the 3 age groups and populations defined by the 2015 HEDIS Controlling Blood Pressure Technical Specification (control defined as blood pressure <140/90 mm Hg.). These organizations also reported their total number of adult patients for each of these years, which collectively represent about 57% of Sonoma County’s overall adult population.

**Figure 2:**
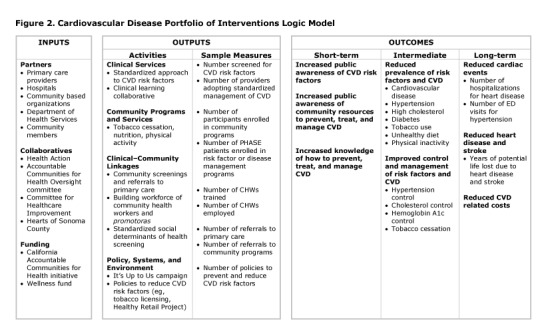
Cardiovascular disease portfolio of interventions logic model, the Hearts of Sonoma County Initiative, Sonoma County, California. Abbreviations: CHW, community health worker; CVD, cardiovascular disease; ED, emergency department; PHASE, Preventing Heart Attacks and Strokes Everyday.

The evaluation of the community engagement efforts — blood pressure screening, CHW outreach, It’s Up to Us media campaign — is a descriptive, process evaluation. Information gathered, both in real time through progress reporting and retrospectively, includes the number of screening events held, number of people screened, number of people with high or very high blood pressure, and number of individuals connected with primary care. There are several community engagement outcomes in which measures are being developed (eg, increased public awareness of CVD risk factors and community resources to help address them) (Figure 2).

**Figure 3:**
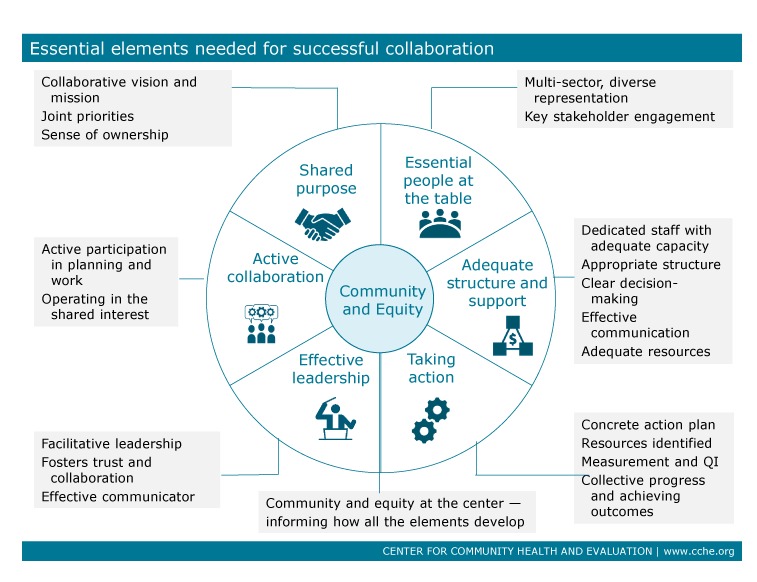
Essential elements needed for effective collaboration, the Hearts of Sonoma County Initiative, Sonoma County, California. Abbreviation: QI, quality improvement.

Details about the evolution of the collaborative structure and process, as well as successes, challenges, and lessons learned, were gathered through document review and interviews with 8 key participants. The data gathering was organized using a framework developed by the Center for Community Health and Evaluation (CCHE) to track key elements in coalition development (Figure 3).

## Results

### Improving clinical care

Four major health systems have participated in the HSC work around implementing the PHASE protocol and other clinic-level interventions. Kaiser Permanente developed the protocol and has implemented it successfully in their 4 Sonoma County clinics. Other health systems have focused initially on pilot implementation in selected clinics or pods within clinics (eg, a large St. Joseph Health Medical Group practice in Santa Rosa). The community health centers began implementing PHASE in 2011 through a Kaiser Permanente Northern California Community Benefits program grant to the Redwood Community Health Coalition. Progress to date has included identification of nearly 25,000 patients with a diagnosis of hypertension, diabetes and/or atherosclerotic CVD across 22 clinic sites. Since baseline of March 2017, community health centers have demonstrated aggregate performance improvements in lifestyle measures including body mass index, tobacco, and depression screenings with documented follow-up plans as well as on prescription measures, including angiotensin converting enzyme/angiotensin receptor blocker and statin prescription rates, among patients aged 55 through 75 with diabetes.

**Figure 4:**
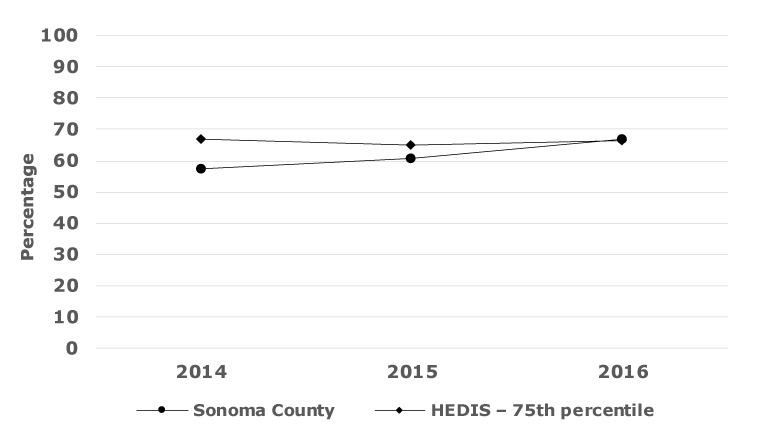
Percentage of hypertension patients aged 18 to 59 years with controlled blood pressure, the Hearts of Sonoma County Initiative, Sonoma County, California.

County-level trends in CVD outcomes assessed by using the shared data from the 4 participating health systems have been encouraging. Figure 4 shows trends in blood pressure control for ages 18–59 years; results were similar in other age groupings. All of the year-to-year changes were significant (*P* < .001), increasing from 58% of participants who had their blood pressure controlled in 2014 to 67% in 2016. HEDIS benchmark trends for a comparable measure essentially did not change during that same period.

### Community engagement

Between 2017 and 2019, the community engagement effort has conducted 99 outreach events, reaching 1,751 individuals, and conducted 1,729 blood pressure screenings. A total of 441 of the people screened were found to have high or very high blood pressure readings and were contacted for follow-up by bilingual Center for Well-Being staff to evaluate the effectiveness of the screening and to motivate them to connect with their doctor or a referred provider. Partners such as St. Joseph Health have integrated It’s Up to Us into their community-based screenings, with staff adapting the campaign to meet the needs of the populations they serve. Future outreach opportunities being explored include senior centers, school parent groups, and grocery stores located in low-income neighborhoods. Work is underway to launch a blood pressure clinic with Santa Rosa Community Health’s Fiesta site, piloting a faster point of entry to care for residents out of care found to have high or very high blood pressure readings. The Center for Well-Being and Santa Rosa Community Health are looking to expand the pilot to other clinic sites in the future.

### Collaborative impact, sustainability, and success factors

The HSC collaborative provides the overall structure and support for the clinical and community activities, including forming relationships for interventions linking clinics and communities. The collaborative has achieved sustainability, a result that eludes many collaboratives. Understanding why it has been successful may provide lessons for other similar collaboratives.

Figure 3 summarizes the 6 elements in the coalition model that were used to enumerate and understand the success of the collaborative. Quotes were drawn from interviews with key HSC stakeholders, which included someone from each of the major participating organizations. HSC successfully fulfilled all 6 of the essential elements in the model. The shared purpose of reducing CVD risk through clinical and community approaches used was agreed to by all, and the language was revisited and updated as the initiative continued. A component of success was a strategic approach in aligning the existing goals, interests, and requirements of individual primary care organizations with the shared communitywide goal of improving CVD health. For example, all of the primary care organizations are evaluated on HEDIS or HEDIS-like measurements. Measurements were developed that would most closely match the specifications of required performance metrics to take advantage of data the organizations were already collecting. This approach and alignment meant that improvements resulting from the collaborative work of the HSC initiative translated to improved outcomes on performance measures that are important to the individual entities participating in the initiative, which in turn supported ongoing investment in the process.

The essential people and organizations were generally present within HSC although several informants noted the absence of community residents to provide a consumer perspective: “We’re struggling with having resident involvement . . . [for example] neighborhood organizations.” Community is at the center of the coalition model to emphasize that the efforts are ultimately designed to improve the health of community residents, who should therefore be engaged to define what matters to them in the way of health and how their health can be improved. The It’s Up to Us campaign is working to increase the level of community engagement.

Effective leadership of HSC has generally been present in the form of a rotating group of clinical leaders from the different health systems. Several informants noted that leadership has come from many of the participating organizations: “Yes, we have strong leaders from all sectors — public health, clinical providers, and community-based organizations.”

Informants were unanimous in praising the staff person from the Department of Health Services for providing more than adequate staffing and support, leveraging the small amount of county funding and the CACHI grant to support the growing number of HSC activities: “Our health department [Department of Health Services] has provided consistent critical support. They have competing priorities but have always been engaged in this effort.”

Active collaboration is a critical but hard-to-define property of effective collaboratives: people and organizations set aside their more narrow organizational interests in support of the whole group. All of the informants understood the concept and agreed that over time people had seen the value of collaboration. As one informant said, “The tone of the meetings is sharing what works so that others can benefit from it. How can we improve care for all, recognizing differences and helping each other. It’s in the nature of how the organization came about. We’re charged with improving quality of life in a number of domains.”

Finally, taking action occurred initially in the form of health care organizations bringing back what they learned from the collaborative to their own organizations to be implemented: “Moving quickly to action to demonstrate value has been important; for example, one of the medical groups implemented a team-based care pilot in her own practice based on their HSC experience. People take things back.” The value of those early learnings helped catalyze other activities, including CACHI and community engagement.

In addition to validating the elements of the CCHE model, we asked respondents in a more open-ended way about the key accomplishments of HSC and why they thought the effort had lasted. They did not often mention specific activities or clinical improvements, but rather that they appreciated the collaboration itself and being able to step outside the competitive realm of their different health systems to focus on what could be done to improve patient and community health. The dialog and shared learnings at the monthly meetings built trust and promoted the active collaboration. Respondents attributed the sustainability of the HSC effort to the building of that level of trust.

## Implications for Public Health

Although the positive results — early but encouraging countywide trends in blood pressure control and significant community engagement activities with more in the works — are important, another goal of the HSC evaluation was to understand the factors behind the staying power and impact of the collaborative. We looked in particular for structural or process factors that might be generalizable to other, similar collaboratives. Three such factors that emerged were starting small and focused, while working within the framework of a larger effort, and providing backbone support that was open-ended and not limited by funding time constraints.

**Start small and focused to build trust and demonstrate value.** The initial seeds of the HSC initiative were the activities of the Committee for Healthcare Improvement (CHI), operating as part Health Action starting in 2007. A small number of clinical champions from the key health organizations came together to see whether sharing lessons from others could benefit their own organizations. They were able to agree on a purpose and mission and move to action fairly quickly even though resources to implement whatever changes they identified were limited and had to come from within their own organizations. These early successes helped build trust and demonstrate the value of the collaborative.

**Operate within a larger structure.** Although the health care work involved a small number of people with a narrow focus, it was embedded in the larger Health Action collaborative. This had 3 long-term advantages. First, leaders on the Health Action Council approved projects undertaken by CHI, including HSC, which translated into a leadership and organizational commitment to HSC. Second, connections were created with a larger group of member organizations who were potential collaborators as the work grew in scope. Third, it was easier to secure long-term backbone support from Sonoma County, because the effort had a broad focus and therefore a wider political constituency.

The lessons about starting small but operating within a larger structure suggest a path for others seeking to ultimately create a large-scale collaborative to achieve health system transformation. Create a large, ambitious collaborative structure and membership, but be willing to focus initial activities narrowly where progress can most readily be made. This requires accepting modest results in terms of health impact, which can also help build the trust required for sustainability. 

Other lessons were learned through this process. Grant-funded collaboratives are often time-limited, and it can be challenging to find funding streams to sustain the effort. A key to the success of HSC was the long-term in-kind support provided by the Sonoma County Department of Health Services. This was enough to provide support to the early focused efforts of HSC. Also, administrative and especially clinical leadership in each organization is essential to teach colleagues, guide the direction of change, and encourage the use of protocols. These can all be difficult for clinicians to accept and implement, so leadership is essential. Finally it is important to have small successes and celebrate them along the way. This keeps people interested and knowing progress is being made. Having the shared purpose, however, is key. These lessons are consistent with what others have found (8) and not revolutionary, but they are often ignored in the sense of urgency created by the need to transform the health care system and the availability of large-scale, but time-limited, funding available through State Innovation Model grants (3), Medicaid DSRIP (Delivery System Reform Incentive Payment) Waivers (13), and other sources.

Some limitations should be noted. The evaluation of the community engagement activities has been a more qualitative, process evaluation; longer-term outcome measures are still being developed. The data on CVD outcomes (eg, blood pressure) are limited to the 4 participating providers, which represent just over half of the county patient population. Finally, HSC is focused on CVD only, which, although a leading cause of illness and death, is not indicative of overall health system transformation. However, many of the issues that arise in working in CVD (eg, data sharing, collaboration across systems, linking with community resources) are present in broader transformation efforts, so the HSC lessons should apply.

The HSC collaborative members continue to work together. On the horizon are the continued expansion of the community engagement work, the creation of a clinical population health improvement collaborative to broaden and standardize the clinical improvement work, and additional population-level metrics to judge the impact. The goal continues to be implementing targeted, coordinated clinical, community, and policy interventions to improve cardiovascular health, recognizing that only by sustaining efforts over the long term can sustained health improvement be achieved.
